# The effectiveness of high-intensity interval training on cardiometabolic outcomes in middle-aged and elderly populations with chronic diseases: a systematic review and meta-analysis

**DOI:** 10.3389/fphys.2025.1669941

**Published:** 2025-10-29

**Authors:** Qiang Li, Gang Xu, Chunai Liu, Lina Gao, Hongli Yu

**Affiliations:** 1 School of Physical Education, Shangrao Normal University, Shangrao, China; 2 School of Physical Education, Sichuan University of Science & Engineering, Zigong, China; 3 School of Education, Chuxiong Normal University, Chuxiong, China; 4 Department of Economics and Trade, Deyang Vocational College of Technology and Trade, Deyang, China

**Keywords:** high-intensity interval training, cardiometabolic outcomes, middle-aged and Elderly populations, chronic diseases, systematic review, meta-analysis

## Abstract

**Objective:**

To assess the effectiveness of High-Intensity Interval Training (HIIT) on cardiometabolic outcomes in Middle-Aged and Elderly Populations (MAEP) with chronic diseases.

**Methods:**

Four databases (PubMed, Cochrane Library, Embase, and Web of Science) were searched from inception to May 30, 2025. Software package RevMan version 5.4 and Stata 18 were conducted to determine publication bias and randomized controlled trials (RCTs) exploring the impacts of HIIT to components of high-density lipoprotein cholesterol (HDL-C), systolic blood pressure (SBP), diastolic blood pressure (DBP), fasting blood glucose (BG), and triglyceride (TG) on MAEP with chronic diseases. Subgroup moderator analyses were conducted based on the intervention duration and geographic region.

**Results:**

Out of 6,106 studies, 21 RCTs involving 1,066 participants were included. HIIT significantly benefits for DBP (SMD = −0.23, 95% CI: −0.39 to −0.08, p < 0.01), HDL-C (SMD = 0.41, 95% CI: 0.11 to 0.71, p < 0.01), TG (SMD = −0.68, 95% CI: −1.20 to −0.16; p < 0.05) and BG (SMD = −0.37, 95% CI: −0.69 to −0.06; p < 0.05), However, HIIT did not significantly reduce SBP (SMD = −0.14, 95% CI: -0.38 to 0.11, p > 0.05) among MAEP with chronic diseases. Subgroup analyses suggested that HIIT protocols with intervention duration and geographic region significantly reduced heterogeneity for outcomes such as SBP and HDL-C.

**Conclusion:**

While HIIT did not significantly reduce SBP, it yielded meaningful benefits for DBP, HDL-C, TG, and BG in MAEP with chronic diseases. The findings suggest that the effectiveness of HIIT may vary by region and intervention duration, highlighting the importance of tailoring HIIT protocols to specific populations and contexts.

**Systematic Review Registration:**

identifier CRD420251063576.

## 1 Introduction

Cardiometabolic diseases, such as dyslipidemia, type 2 diabetes, hypertension, and cardiovascular conditions, continue to represent the foremost contributors to worldwide morbidity and death, with their burden most pronounced among middle-aged and elderly populations (MAEP). With the global demographic shift toward aging societies, the prevalence of chronic diseases is projected to rise further, creating profound challenges for healthcare systems and generating substantial economic and societal burdens (Bloom et al., 2015). Consequently, there is an urgent need for effective, feasible, and sustainable interventions to mitigate cardiometabolic risk in these populations.

Lifestyle interventions, including physical activity, have gained traction as non-pharmacological strategies to mitigate cardiometabolic risk, with high-intensity interval training (HIIT) emerging as a particularly promising modality. HIIT, defined by successive brief episodes of intense exercise alternated with low-intensity recovery intervals, has been shown to produce significant enhancements in aerobic capacity, lipid metabolism, and insulin responsiveness (Gibala et al., 2012). In comparison to conventional moderate-intensity continuous training (MICT), HIIT offers time-efficient benefits that are particularly advantageous for older adults who may be constrained by physical limitations or time barriers (Edwards et al., 2023). However, concerns regarding the safety, feasibility, and long-term adherence to HIIT in populations with chronic conditions remain unresolved (O'Brien et al., 2020).

Recent systematic reviews and meta-analyses have demonstrated the effectiveness of HIIT in improving various cardiometabolic parameters in specific clinical populations, notably, beneficial effects have been observed among individuals diagnosed with conditions such as type 2 diabetes and heart failure (Álvarez et al., 2018; Yue et al., 2022). For instance, Jelleyman et al. (2015) conducted a 50 studies included meta-analysis and reported that HIIT produced significant enhancements in glucose metabolism and reduce insulin resistance in individuals with or at risk of type 2 diabetes mellitus (Jelleyman et al., 2015). Similarly, Weston et al. (2014) found that HIIT was associated with more pronounced enhancements in VO_2_max and metabolic profiles compared to MICT among individuals with lifestyle-related cardiometabolic conditions (Weston et al., 2014). In cardiac rehabilitation populations, HIIT has also demonstrated encouraging outcomes in terms of augmenting cardiovascular function and enhancing patients’ quality of life. Hannan et al. (2018) reported in their systematic review that HIIT was not only safe but also more effective than MICT in enhancing aerobic capacity among cardiac patients (Hannan et al., 2018). Furthermore, Ramos et al. (2015) demonstrated that HIIT could significantly improve vascular endothelial function, a key predictor of cardiovascular events, particularly among hypertensive and obese individuals (Ramos et al., 2015).

Despite these encouraging findings, the evidence base for middle-aged and elderly populations remains limited and inconsistent. The aging process is commonly linked to diminished physiological resilience, decreased skeletal muscle mass, impaired glucose metabolism, and lower exercise tolerance, all of which may influence the efficacy and safety profile of high-intensity physical training (Seals et al., 2016). Some studies suggest that older adults can derive similar cardiometabolic benefits from HIIT as younger counterparts (Keating et al., 2017), while others raise concerns about adherence, injury risk, and the need for individualized protocols (Milanović et al., 2015). Moreover, considerable heterogeneity exists in HIIT protocols across studies, including variations in intensity, interval duration, frequency, and intervention length, which complicates direct comparisons and limits the generalizability of findings (Maillard et al., 2018). In particular, studies targeting individuals over 45 years of age with multiple chronic conditions remain underrepresented in current meta-analyses.

Given these gaps, there is a critical need to systematically synthesize the available evidence on the cardiometabolic effects of HIIT in MAEP with chronic diseases. Therefore, the present systematic review and meta-analysis aims to evaluate the effectiveness of HIIT in improving key cardiometabolic outcomes in this underexplored population, thereby providing an evidence base to inform clinical recommendations and exercise prescription guidelines.

## 2 Materials and methods

### 2.1 Protocol

The methods section was designed to ensure maximal transparency. Specifically, this study detailed the search strategy, screening procedures, risk of bias assessment, and statistical methods, including heterogeneity analysis, sensitivity tests, and subgroup approaches, to strengthen reproducibility. The methodology employed in this study aligns with the guidelines established by the Preferred Reporting Items for Systematic Reviews and Meta-Analyses for Meta-Analyses (PRISMA-MA), which is widely recognized in clinical and medical research. In addition, the procedures conform to the methodological recommendations established by the Cochrane Collaboration. These frameworks have been extensively described in prior foundational literature (Higgins and Green, 2011; Page et al., 2021). To promote transparency and replicability, a completed PRISMA-MA checklist is included in Supplementary Table 1. Moreover, the research protocol was registered in advance with the PROSPERO database and is accessible under the corresponding registration number [CRD420251063576], ensuring compliance with best practices for systematic review reporting and protocol documentation.

### 2.2 Search strategy and study selection

A rigorous literature search was conducted independently by two researchers across four prominent electronic databases, which are PubMed, Cochrane Library, Embase, and Web of Science. This search encompassed all relevant publications from each database’s inception through May 30, 2025. To ensure methodological rigor, the search strategy was structured according to the PICOS criteria, defining Population, Intervention, Comparator, Outcomes, and Study Design, as detailed in Supplementary Material 1–4. The review specifically targeted studies involving MAEP with chronic health conditions, where HIIT served as the primary intervention in comparison to standard care. Cardiometabolic outcomes under scrutiny included high-density lipoprotein cholesterol (HDL-C), systolic blood pressure (SBP), diastolic blood pressure (DBP), fasting blood glucose (BG), and triglyceride (TG), These variables were selected based on their consistent use as primary endpoints in previous HIIT intervention studies targeting chronic disease populations and their strong clinical relevance to cardiovascular and metabolic health (Wewege et al., 2017). Only randomized controlled trials (RCTs) were considered eligible for inclusion as part of the study design criteria. The search strategy incorporated keywords in conjunction with Boolean operators to refine the retrieval process: (Middle aged OR Middle-aged OR elderly OR older adult* OR senior* OR geriatric OR old* OR aging OR frail elderly) AND (chronic disease OR comorbid* OR multimorbid* OR long term condition* OR noncommunicable disease* OR NCD OR cardiovascular disease* OR CVD OR coronary artery disease OR CAD OR hypertension OR metabolic syndrome OR diabetes mellitus OR type 2 diabetes OR T2DM OR dyslipidemia* OR obesity OR overweight) AND (high intensity interval training OR HIIT OR sprint interval training OR SIT OR interval train* OR high intensity intermittent exercise OR anaerobic interval train*) AND (RCT OR randomized controlled trial). The complete research approach is available in Supplementary Table 2. This study also searched gray literature sources, including conference proceedings and trial registries (e.g., ClinicalTrials.gov, WHO ICTRP), to minimize publication bias. However, very few eligible trials were identified outside of peer-reviewed journals. To maximize the comprehensiveness of the evidence base, an additional manual search was conducted to identify relevant systematic reviews, meta-analyses, major international conference proceedings, and reference lists of the included studies. Two independent reviewers (QL and GX) conducted the initial screening of study titles and abstracts, with their identities anonymized to mitigate potential bias during the selection process. Inter-rater agreement between the two reviewers was quantified using Cohen’s kappa (κ = 0.84), indicating strong consistency in study selection decisions (Cohen, 1960). Endnote software (version 21, Thompson ISI ResearchSoft) was employed to facilitate the organization of citations and to efficiently detect and eliminate duplicate entries, thereby improving the reliability of the dataset. Subsequently, the same two reviewers independently conducted a blinded assessment of the full-text articles. In cases where disagreement arose regarding study inclusion, resolution was achieved either through mutual consensus or, if needed, by involving a third reviewer (HL).

### 2.3 Inclusion and exclusion criteria

The criteria for study eligibility in the meta-analysis were determined by assessing key factors, including population characteristics, intervention type, comparator conditions, outcome measures, and study design. The inclusion criteria encompassed the following:

1. MAEP, which aged 45 years and above (WHO, 2025), classified as chronic diseases, without other coexisting medical conditions.
2. Interventions involving HIIT, which includes various forms of exercise such as 4 × 4 min pedalling, walking and running, delivered in person.
3. Standard care will be the comparisons.
4. Availability of health outcome data.
5. RCTs, whether employing parallel or crossover frameworks, were included without imposing limitations based on criteria such as chronic disease classification, geographic region, ethnic background, language, or publication date. Existing scholarly definitions were leveraged to classify interventions as mHealth in-person, and eHealth approaches.

Exclusion criteria included: 1) researches that included participants other than human subjects; 2) interventions other than HIIT; 3) unavailable data; and 4) protocols or studies not employing RCTs.

No restrictions were placed on intervention duration at the design stage to maximize the inclusiveness of evidence. While this enabled capture of a wide range of HIIT protocols, it also introduced variability in exposure length, which we acknowledge as a limitation of the present study design. Although not all included participants had diagnosed hypertension, SBP and DBP were included as outcomes due to their established role as clinical risk markers across multiple chronic conditions. The rationale was to evaluate whether HIIT could serve as a preventive or therapeutic tool for blood pressure regulation even in populations without hypertension at baseline.

### 2.4 Data collection and management

Two reviewers independently carried out the extraction of data from the eligible RCTs, each operating under blinded conditions to mitigate potential bias. To ensure uniformity and reliability in the recording process, a standardized extraction template was utilized. This form had been developed based on the recommendations provided by the Cochrane Consumers and Communication Review Group (Chandler et al., 2019). One included study de Matos et al. (2021) reported sex-stratified outcomes for men and women (de Matos et al., 2021) F and (de Matos et al., 2021) M undergoing high-intensity interval training. To improve the precision of pooled estimates and to explore sex-specific effects, we treated these two subgroups as independent data entries in the meta-analysis. Subgroup definitions were as follows: intervention duration was categorized as short-term (0–8 weeks), medium-term (9–16 weeks), and long-term (17–24 weeks). Geographic region was classified into European and non-European categories. These definitions were based on prior literature conventions and allowed for sufficient distribution of studies across subgroups to ensure meaningful comparison. This approach has been previously recommended when subgroup data are clearly reported and do not involve statistical dependence (Borenstein et al., 2021).

The compiled dataset encompassed four major domains:

1. Essential publication details, including the first author’s name, the geographical location in which the study was carried out and the corresponding year it was published;
2. Participant demographic characteristics, such as the age range of subjects, the defined population under study, and total sample size;
3. Intervention allocation information, detailing group assignments (intervention vs. control), a comprehensive description of the intervention protocol, and the conditions maintained for the control group;
4. Physical activity-related variables, covering type of exercise, training duration, session frequency, and total cycle length.

In parallel, general characteristics of the eight selected meta-analyses were also extracted by two independent reviewers using the same standardized form (Higgins et al., 2021). Any discrepancies encountered during the data collection phase were resolved through discussion, and where consensus could not be reached, a third reviewer was consulted for adjudication.

### 2.5 Quality appraisal

The methodological quality appraisal of the selected RCTs was performed using the Cochrane Collaboration’s Risk of Bias (ROB) assessment tool, following the methodological instructions detailed in reference (Shuster, 2011). Two independent reviewers carried out the assessment for each trial. As specified in the Cochrane Handbook version 5.1.0 (Higgins et al., 2011), the evaluation focused on identifying potential sources of bias by examining seven predefined domains:1) Generation of the random sequence, aimed at evaluating selection bias; 2) Concealment of allocation, also targeting selection bias; 3) Blinding of participants and study personnel, addressing performance bias; 4) Blinding of outcome assessors, which relates to detection bias; 5) Management of incomplete outcome data, pertinent to attrition bias; 6) Selective outcome reporting, reflective of reporting bias; 7) Any additional sources of potential bias not captured by the previous categories (Collaboration, 2012).

Each study was classified into one of three risk levels, low, unclear, or high, based on these criteria. The initial assessment was undertaken separately by two reviewers. In cases where inconsistencies arose, resolution was achieved through discussion, and if consensus could not be reached, a third reviewer was consulted. The entire risk of bias assessment was implemented utilizing RevMan software (version 5.4, The Cochrane Collaboration, 2020; Collaboration, 2020).

Furthermore, to address potential concerns regarding high risk of bias, we explicitly noted that approximately 30% of included studies demonstrated high risk in at least one ROB domain. Although all studies were retained to preserve statistical power and ensure sufficient coverage across outcomes, we conducted sensitivity analyses by systematically excluding high-risk trials. These analyses demonstrated that the overall direction and statistical significance of the pooled results remained unchanged, supporting the robustness of our findings. However, we acknowledge that the inclusion of high-risk studies may introduce uncertainty, and thus, the conclusions should be interpreted with caution.

### 2.6 Statistical analysis

All statistical computations were conducted utilizing Stata version 18 to synthesize effect sizes and assess potential biases across studies. Due to variability in how depression-related outcomes were measured among the included trials, effect sizes were uniformly transformed to enhance comparability and accuracy. Specifically, data were standardized using standardized mean difference (SMD) and 95% confidence intervals (CI) (Higgins et al., 2003; Sterne, 2009). The SMD was derived by calculating the difference between post-intervention and pre-intervention means, followed by normalization using the pooled standard deviation (SD) measured at the study endpoint (Raudenbush, 1984). This approach enables the comparison of studies utilizing different measurement scales by adjusting for discrepancies in unit definitions (Rosenthal and Jacobson, 1968).

To quantify heterogeneity, I^2^ statistics were employed. The interpretation of I^2^ values followed conventional thresholds: 1) An I^2^ between 0% and 25% was considered to indicate low heterogeneity, implying minimal variability among the included studies. 2) Values from 25% to 50% represented moderate heterogeneity, suggesting a moderate degree of inconsistency that warranted exploration of potential influencing factors. 3) An I^2^ range of 50%–75% denoted substantial heterogeneity, highlighting notable differences likely attributable to variations in study methodologies, participant characteristics, or intervention types. 4) When I^2^ exceeded 75%, considerable heterogeneity was inferred, necessitating cautious interpretation of the aggregated results due to possible underlying biases or confounding elements. To enhance precision, 95% confidence intervals for I^2^ statistics were calculated using non-central chi-square approximation methods, as recommended in recent meta-analysis guidelines. To evaluate the risk of publication bias, funnel plot analysis was conducted as part of the diagnostic process.

For outcomes such as SBP and DBP, where all studies used the same unit (mmHg), weighted mean differences (WMD) could theoretically be reported. However, due to variation in baseline values and reporting methods across trials, we opted for SMD to harmonize effect size estimation across outcomes. This decision ensures comparability and robustness in pooled analyses, but we acknowledge it as a methodological limitation.

## 3 Results

### 3.1 Search results and study characteristics

**FIGURE 1:**
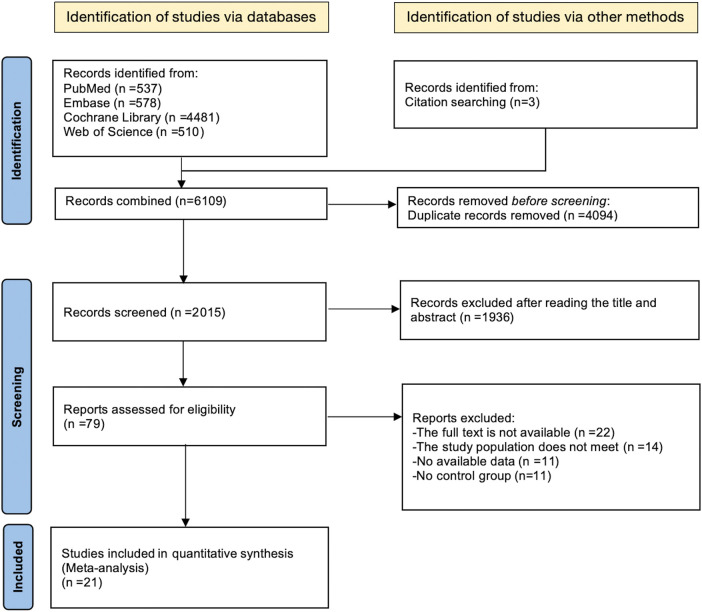
PRISMA diagram depicting the sequential steps of the selection process.

The process of literature identification and selection has been outlined in the PRISMA-MA flowchart (Figure 1). An initial comprehensive search across four electronic databases retrieved a total of 6,106 records, with an additional three studies identified through manual citation tracking. After removing 4,049 duplicate entries, 2,015 records were excluded during the preliminary screening of titles and abstracts. This left 1,936 articles for full-text evaluation based on the predefined eligibility criteria.

Following a thorough review of the full-text articles, a total of 79 studies were excluded for the specific reasons: 11 researches lacked sufficient outcome data; 22 articles did not provide compatible study design; 14 reports failed to meet the inclusion standards; A valid control group was absent in 11 of the included trials.

Consequently, 21 RCTs met the inclusion criteria and were incorporated into the meta-analysis, involving 1,066 participants, all classified as MAEP with chronic diseases. Among them, 634 individuals were assigned to the intervention group, while 432 participants served as controls. The ages ranged from 46 to 67 years for participants.

Geographically, the included researches represented a diverse set of countries and regions, including Norway and Spain from Europe; the United States, Colombia, and Brazil from the Americas; China and South Korea from Asia; as well as Australia from Oceania. All included studies were published in English and were designed as RCTs published up to May 2025. A comprehensive summary of participant demographics and intervention characteristics is provided in Supplementary Table S3.

### 3.2 Quality evaluation

**FIGURE 2:**
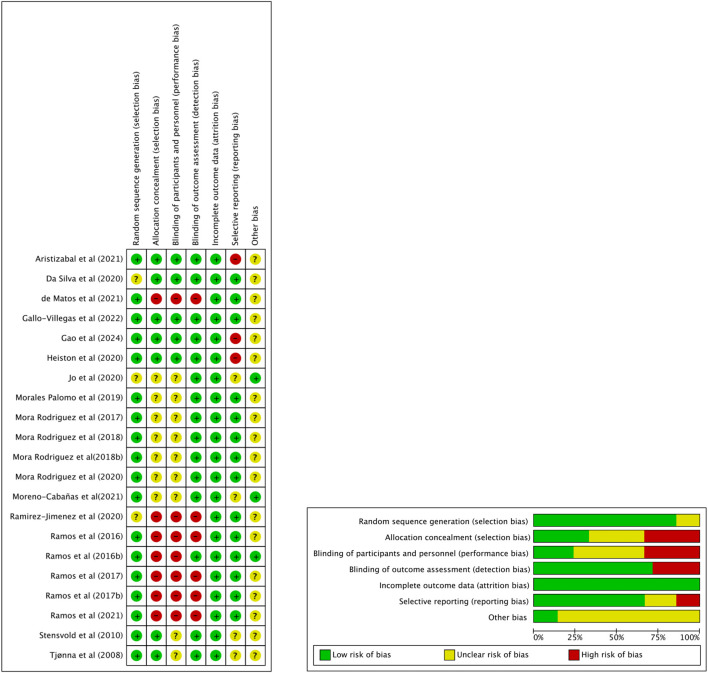
Risk of Bias graph and summary.

ROB was evaluated using the Cochrane Risk of Bias tool, as shown in Figure 2. Most studies (77.3%) demonstrated low risk in random sequence generation, including Aristizabal et al. (2021) and Gallo-Villegas et al. (2022). However, allocation concealment was more variable, with only 40.9% of studies rated low risk, while 31.8%, such as de Matos et al. (2021) and Ramirez-Jimenez et al. (2020), showed high risk. Performance bias was common: 36.4% of studies (e.g., Ramos et al., 2016) were rated high risk regarding blinding is often not feasible in exercise intervention studies. Outcome assessor blinding was more consistent, with 68.2% of studies judged as low risk.

Attrition bias was well controlled, with 95.5% of studies showing low risk. For selective reporting, 68.2% were rated low risk, although two studies (Heiston et al., 2020; Aristizabal et al., 2021) were rated high. Other bias was mostly unclear across studies (81.8%), reflecting insufficient reporting. Overall, while the quality of included trials was generally acceptable, limitations in allocation concealment and performance blinding warrant cautious interpretation of pooled results.

### 3.3 Meta-analysis

#### 3.3.1 Effect of HIIT on SBP

**FIGURE 3:**
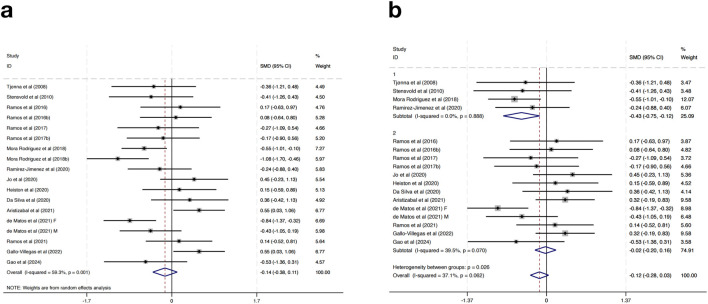
**(a)** Overall and **(b)** subgroup meta-analysis of the effect of HIIT on SBP.

Seventeen studies were included in the meta-analysis examining the effects of HIIT on SBP in MAEP with chronic diseases. The overall pooled result using a random-effects model showed that HIIT did not significantly reduce SBP compared to control groups (SMD = −0.14, 95% CI: -0.38 to 0.11, *p* > 0.05), and moderate heterogeneity was observed (I^2^ = 59.3%, *p* = 0.001) (Figure 3a).

To explore sources of heterogeneity and potential differential effects, subgroup analyses were performed based on geographic region. In studies conducted in European countries (n = 4), HIIT was associated with a statistically significant reduction in SBP (SMD = −0.43, 95% CI: -0.75 to −0.12, *p* < 0.01), with no observed heterogeneity (I^2^ = 0.0%, *p* = 0.888). Conversely, non-European studies (n = 13) showed no significant change in SBP (SMD = −0.02, 95% CI: -0.20 to 0.16, *p* > 0.05), although heterogeneity decreased (I^2^ = 39.5%, *p* = 0.070). A statistically significant difference was found between subgroups (*p* = 0.026), indicating that regional factors may contribute to the variation in intervention effects (Figure 3b). Further subgroup analyses based on intervention duration, training frequency, or session length did not result in significant changes or reduce heterogeneity, suggesting that these factors alone may not fully explain the differential responses.

Taken together, these findings suggest that while HIIT does not show a significant overall effect on reducing SBP in MAEP with chronic diseases, it may offer modest benefits in certain populations, such as those in European settings. The lack of consistent blood pressure reduction limits the generalizability of HIIT as a stand-alone intervention for SBP management in this population. However, its potential for improving other cardiometabolic outcomes should still be considered within a broader context of multimodal interventions.

#### 3.3.2 Effect of HIIT on DBP

**FIGURE 4:**
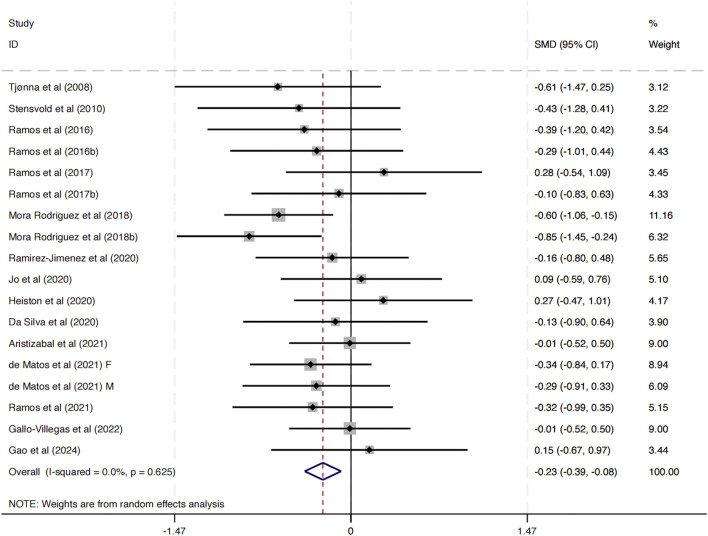
Meta-analysis of the effect of HIIT on DBP.

This study included 17 investigations examining the impact of HIIT on DBP. The combined analysis, conducted with a random-effects model, revealed that HIIT interventions resulted in a statistically significant decrease in DBP compared to control conditions (SMD = −0.23, 95% CI: -0.39 to −0.08, *p* < 0.01) (Figure 4). Importantly, the heterogeneity across studies was negligible (I^2^ = 0.0%, *p* = 0.625), indicating a high level of consistency in the direction and magnitude of the effect across different study populations, intervention protocols, and geographic locations.

These findings suggest that HIIT is associated with a modest but statistically significant improvement in diastolic blood pressure among MAEP with chronic diseases. Given the role of elevated DBP in cardiovascular risk, these results provide supportive evidence for incorporating HIIT as a non-pharmacological strategy to manage blood pressure in this population.

#### 3.3.3 Effect of HIIT on HDL-C

**FIGURE 5:**
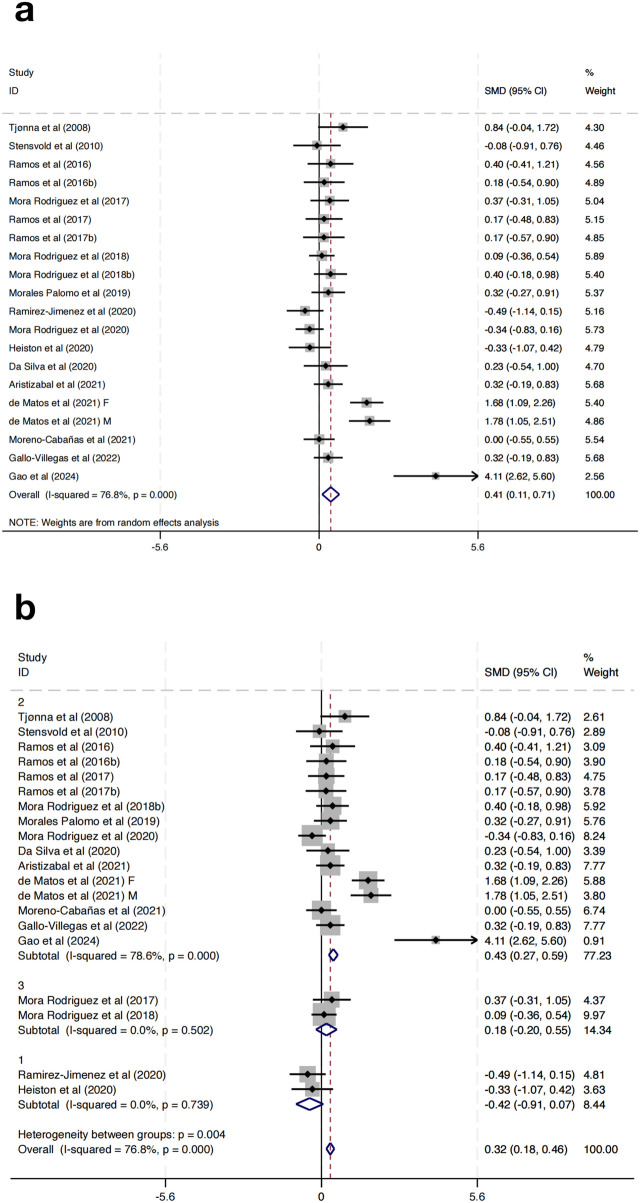
**(a)** Overall and **(b)** subgroup meta-analysis of the effect of HIIT on HDL-C.

Twenty studies were incorporated to evaluate the impact of HIIT on HDL-C in MAEP with chronic diseases. The pooled SMD demonstrated a statistically significant enhancement in HDL-C following HIIT (SMD = 0.41, 95% CI: 0.11 to 0.71, p < 0.01). Nevertheless, substantial heterogeneity was detected among the included studies (I^2^ = 76.8%, p < 0.001) (Figure 5a).

To investigate possible contributors to heterogeneity, subgroup analyses were stratified according to the length of the intervention period: Group 1 (0–8 weeks), Group 2 (9–16 weeks), and Group 3 (17–24 weeks). The subgroup analysis revealed that the heterogeneity was substantially reduced within subgroups (Group 1: I^2^ = 0.0%, p = 0.739; Group 2: I^2^ = 78.6%, p < 0.001; Group 3: I^2^ = 0.0%, p = 0.502), indicating that intervention duration may be a significant source of heterogeneity (Figure 8). Notably, Group 2 (9–16 weeks) showed a significant effect (SMD = 0.43, 95% CI: 0.27–0.59), while Group 1 (SMD = −0.42, 95% CI: −0.91 to 0.07) and Group 3 (SMD = 0.18, 95% CI: −0.20–0.55) did not reach statistical significance (Figure 5b). More importantly, subgroup analyses based on training frequency, geographic region, or session length did not result in significant changes or reduce heterogeneity, suggesting that these factors alone may not fully explain the differential responses. Additionally, the test for subgroup differences indicated a significant intergroup difference reaching statistical significance (p = 0.004), further supporting the impact of intervention duration on the overall outcomes.

To further explore heterogeneity, this study classified HIIT protocols into subtypes (sprint interval training, short-interval HIIT, long-interval HIIT) based on work-to-rest ratio, interval duration, and weekly volume. Exploratory analyses indicated that sprint interval training and short-interval HIIT tended to elicit greater improvements in lipid outcomes, while long-interval HIIT demonstrated more consistent effects on blood pressure regulation. Although the number of trials limited the statistical power of formal meta-regression, this pattern suggests that protocol characteristics are important moderators of response to HIIT.

#### 3.3.4 Effect of HIIT on TG

**FIGURE 6:**
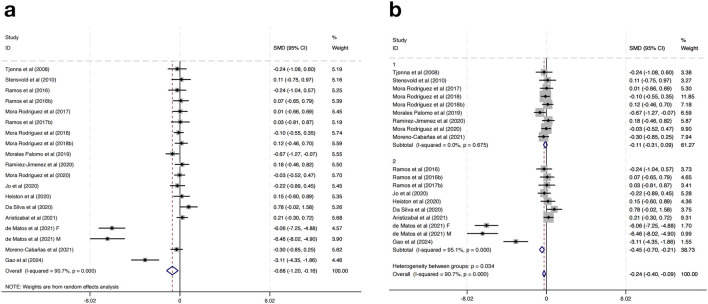
**(a)** Overall and **(b)** subgroup meta-analysis of the effect of HIIT on TG.

Eighteen studies were incorporated to assess the impact of HIIT on TG levels among MAEP with chronic diseases. The pooled effect size revealed a statistically significant reduction in TG levels following HIIT interventions compared to controls, with a SMD of −0.68 (95% CI: −1.20 to −0.16; p < 0.05). Nevertheless, a high degree of heterogeneity was present among the included studies (I^2^ = 90.7%, p = 0.000), indicating considerable variability in effect estimates (Figure 6a).

To explore the potential sources of heterogeneity, a subgroup analysis based on geographical region was conducted. Studies were categorized into two subgroups: those conducted in European countries (Group 1) and those conducted in non-European regions (Group 2). The results showed that in the European subgroup, HIIT had a small and non-significant effect on TG levels (SMD = −0.11, 95% CI: −0.31 to 0.09; I^2^ = 0.0%, p = 0.675), while in the non-European subgroup, HIIT was associated with a significantly greater reduction in TG levels (SMD = −0.45, 95% CI: −0.70 to −0.21; I^2^ = 95.1%, p = 0.000) (Figure 6b). Further subgroup analyses based on intervention duration, training frequency, or session length did not result in significant changes or reduce heterogeneity, suggesting that these factors alone may not fully explain the differential responses.

Importantly, the heterogeneity between the two subgroups was statistically significant (p = 0.034), indicating that the region where the study was conducted may be a contributing factor to the overall heterogeneity. These findings indicate that HIIT may have a more pronounced effect on TG reduction in non-European populations compared to European populations.

#### 3.3.5 Effect of HIIT on BG

**FIGURE 7:**
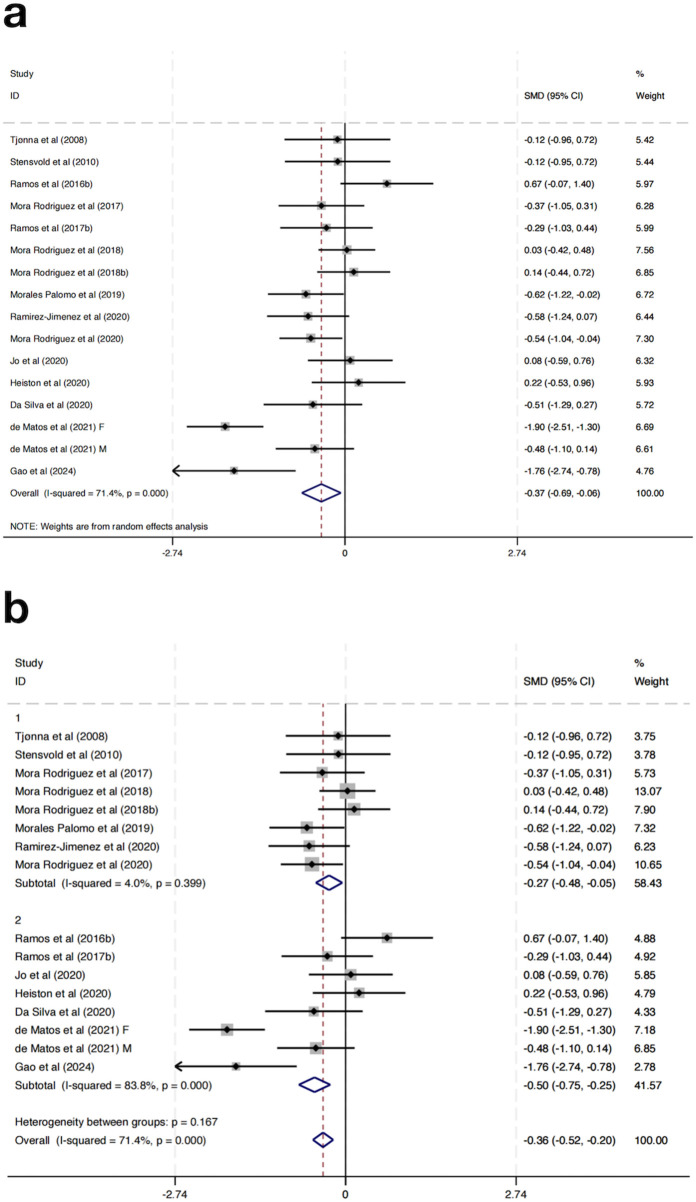
**(a)** Overall and **(b)** subgroup meta-analysis of the effect of HIIT on BG.

Seventeen randomized controlled trials were incorporated to evaluate the impact of HIIT on BG among MAEP diagnosed with chronic diseases. The aggregated results indicated that HIIT led to a statistically significant reduction in BG levels when compared with control conditions, yielding a SMD of −0.37 (95% CI: –0.69 to −0.06; p < 0.05). Nonetheless, considerable heterogeneity was detected across the included studies (I^2^ = 71.4%, p = 0.000), suggesting notable variability in intervention effects among the included trials (Figure 7a).

To explore sources of heterogeneity, a subgroup analysis based on study location was conducted. Studies were grouped into those conducted in Europe (Group 1) and those from non-European regions (Group 2). In the European subgroup, HIIT significantly reduced fasting BG levels (SMD = −0.27, 95% CI: -0.48 to −0.05), with low heterogeneity (I^2^ = 4.0%, p = 0.399). In contrast, the non-European subgroup showed a slightly greater effect size (SMD = −0.50, 95% CI: -0.75 to −0.25), but with considerable heterogeneity (I^2^ = 83.8%, p = 0.000) (Figure 7b). Further subgroup analyses based on intervention duration, training frequency, or session length did not result in significant changes or reduce heterogeneity, suggesting that these factors alone may not fully explain the differential responses.

Although the test for subgroup differences was not statistically significant (p = 0.167), the marked reduction in heterogeneity within the European group suggests that geographical region may partly explain the observed variability in outcomes. These results indicate that HIIT has a beneficial effect on fasting blood glucose across regions, with more consistent results in studies conducted in Europe.

### 3.4 Tests for bias

**FIGURE 8:**
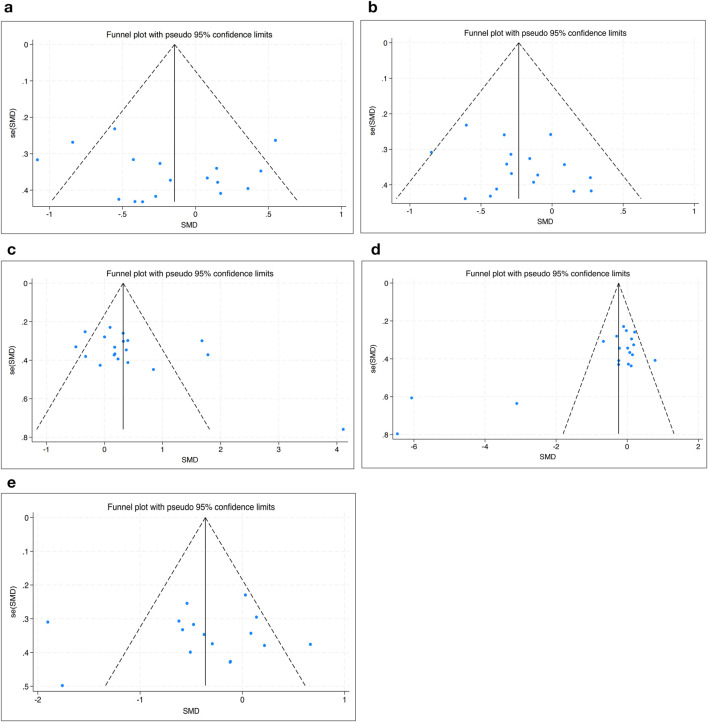
Bias funnel plot of HIIT on **(a)** SBP, **(b)** DBP, **(c)** HDL-C, **(d)** TG, and **(e)** BG.

The funnel plot analysis showed that the scatter points were evenly distributed within the funnel according to the effectiveness of HIIT to SBP, DBP, HDL-C, TG and BG, indicating no publication bias (Figure 8).

### 3.5 Sensitivity analysis

**FIGURE 9:**
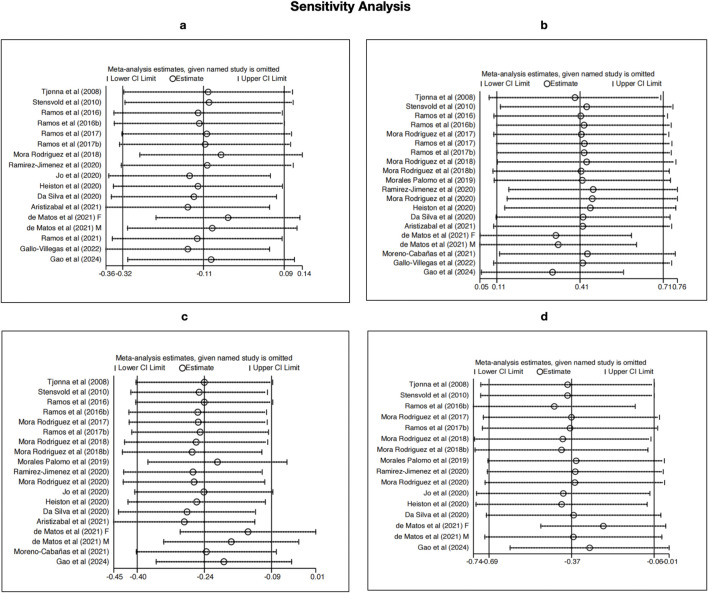
Sensitivity analyses using the leave-one-out method for **(a)** SBP, **(b)** HDL-C, **(c)** TG, and **(d)** BG.

To evaluate the robustness of the pooled effects and identify any influential studies, a leave-one-out sensitivity analysis was conducted for SBP, HDL-C, TG, and BG (Figure 9). The results indicated that no single study substantially changed the direction or significance of the overall effect estimates, suggesting that the findings are generally stable. For SBP, the overall null result remained unchanged across exclusions, though studies such as Aristizabal et al. (2021) and Gallo-Villegas et al. (2022) showed slight influence on the confidence intervals. The results for HDL-C were consistent, with a persistent positive effect across all iterations, supporting the reliability of HIIT’s impact on lipid metabolism.

For TG and BG, the sensitivity analyses confirmed the stability of the significant reductions observed, even though the effect sizes slightly varied when studies like Gao et al. (2024) and Da Silva et al. (2020) were omitted. These results reinforce the credibility of our meta-analysis and suggest that the observed benefits of HIIT on HDL-C, TG, and BG are not driven by any single study, despite the initially high heterogeneity in these outcomes.

## 4 Discussion

This research evaluated the effectiveness of HIIT on cardiometabolic outcomes, including SBP, DBP, HDL-C, TG, and BG, among MAEP with chronic diseases. The findings are derived from 21 RCTs involving over 1,000 participants across multiple geographic regions and clinical populations, offering a comprehensive synthesis of HIIT’s potential therapeutic value in non-pharmacological chronic disease management. This discussion is organized around three key themes: (1) the overall effects of HIIT on cardiometabolic outcomes, (2) sources of heterogeneity and protocol-related moderators, and (3) contextual and clinical implications of findings. This thematic structure allows clearer linkage between empirical results, underlying physiological mechanisms, and practical applications.

The characteristics of the included studies may have contributed to the observed heterogeneity in effect estimates. Notably, the variation in geographic region, intervention duration, and sample characteristics may influence the cardiometabolic response to HIIT. For instance, our subgroup analysis showed that heterogeneity in SBP, TG, and BG outcomes was markedly reduced when studies conducted in European countries were analyzed separately, suggesting that population-specific or contextual factors (e.g., baseline health status, healthcare access, lifestyle norms) may moderate the effects of HIIT. Similarly, when stratifying HDL-C outcomes by intervention duration, the 0–8 weeks subgroup exhibited reduced heterogeneity, which may indicate that shorter intervention periods yield more consistent responses among middle-aged and elderly populations with chronic diseases. Additionally, variations in exercise protocols, such as intensity (%HRmax), frequency (2–5 sessions/week), and exercise mode (cycling, treadmill, running), could have contributed to the outcome variability. For example, some studies implemented protocols with 3–4 intervals at 90% HRmax, while others used more frequent intervals or mixed modalities, which may produce differential physiological adaptations. The heterogeneity observed across outcomes likely reflects both methodological variation and biological diversity. Specifically, differences in supervision, exercise modality (cycling vs. treadmill), and frequency may have influenced adherence and physiological adaptation. Future research should adopt standardized reporting of HIIT parameters to facilitate cross-study comparability and enable more precise recommendations.

Furthermore, sex-specific responses, as explored in the study by de Matos et al. (2021), highlight the importance of demographic considerations in tailoring HIIT interventions for optimal metabolic outcomes. These findings align with previous research emphasizing the moderating effects of intervention context and participant characteristics on HIIT efficacy (Weston et al., 2014; Milanović et al., 2015).

The pooled results demonstrated that HIIT led to discernible reductions in DBP, TG, and BG, as well as an increase in HDL-C. Notably, HIIT’s impact on SBP was not statistically significant when considering all studies together; however, subgroup analysis revealed that European studies showed a significant SBP-lowering effect. These geographic differences suggest that regional contextual factors, such as differences in healthcare infrastructure, exercise adherence, cultural attitudes toward physical activity, or genetic predisposition, may influence responsiveness to HIIT. Regional variation in outcomes may not only reflect differences in HIIT protocol design but also broader cultural and socioeconomic contexts. For example, European trials often reported structured, supervised sessions delivered within healthcare or rehabilitation settings, which may promote higher adherence and compliance. In contrast, non-European trials were more heterogeneous, with variability in supervision, access to facilities, and participant baseline health behaviors, which could partly explain inconsistent results. Cultural norms, availability of qualified trainers, and public health infrastructure likely play a mediating role in shaping both feasibility and effectiveness of HIIT. This is supported by prior meta-analyses indicating that cardiovascular adaptations may vary across ethnic and environmental contexts (Li et al., 2025; Batacan et al., 2017).

From a physiological perspective, specific HIIT characteristics such as shorter recovery intervals or higher relative intensity may preferentially stimulate mitochondrial biogenesis, endothelial shear stress, and insulin sensitivity (Gibala et al., 2012; Jelleyman et al., 2015), but may also pose challenges for elderly individuals with comorbidities. This duality highlights the need for tailoring HIIT prescriptions according to population characteristics and resource availability. HIIT significantly improved DBP with a pooled SMD of −0.23 and no heterogeneity, indicating a robust and consistent benefit across study designs, populations, and regions. This observation is consistent with existing vascular physiology research, which has shown that interval training enhances endothelial function and baroreflex sensitivity, particularly in aging populations (Batacan et al., 2017). In contrast, the non-significant overall SBP effect with moderate heterogeneity suggests that HIIT may not uniformly influence systolic regulation, although subgroup analysis showed that European populations benefitted more. This discrepancy may be related to higher baseline SBP levels, differential medication use, or training program structure in European cohorts (Ramos et al., 2015; Milanović et al., 2015; Khalafi et al., 2022b).

With respect to lipid metabolism, HIIT produced a significant increase in HDL-C (SMD = 0.41), though heterogeneity was high. Subgroup analysis by intervention duration clarified this: 9–16 weeks interventions (Group 2) yielded the most consistent and substantial HDL-C gains (SMD = 0.43), while both shorter (0–8 weeks) and longer (17–24 weeks) interventions showed no statistically significant effect and reduced heterogeneity. This suggests that there is a “time window” within which lipid-related physiological adaptations to HIIT may be optimal, consistent with the findings of Racil et al. (Batacan et al., 2017; Racil et al., 2023). As well as related with a recent systematic review and meta-analysis showing that HIIT improves vascular function across diverse adult cohorts, reinforcing its role in cardiovascular risk reduction (Khalafi and Symonds, 2021).

While our analysis focused on HDL-C, the absence of data on low-density lipoprotein cholesterol (LDL-C) represents a notable limitation in fully evaluating lipid-related cardiometabolic risk. LDL-C is a well-established causal factor in atherosclerosis and cardiovascular disease, and its modulation is a critical therapeutic target in clinical settings (Ference et al., 2017). Prior evidence indicates that HIIT may reduce LDL-C levels, particularly in overweight or obese individuals and those with metabolic syndrome (Maillard et al., 2018). However, due to inconsistent reporting and lack of standardized measurement across included trials, LDL-C was not included in our meta-analysis. Future studies should prioritize the inclusion and standardized assessment of LDL-C to enable a more comprehensive understanding of HIIT’s effects on lipid profiles and overall cardiovascular risk reduction.

For TG, HIIT interventions were associated with significant reductions (SMD = −0.68), though extremely high heterogeneity was observed. Subgroup analysis by region revealed that the effect was substantially larger in non-European populations (SMD = −0.45) compared to European populations (SMD = −0.11), where the effect was not statistically significant. These findings support the hypothesis that diet, baseline TG levels, and metabolic phenotype may interact with exercise response (Solera-Martinez et al., 2021). It was similar with the research performed a systematic review and meta-analysis in adults with type 2 diabetes, reporting that chronic HIIT led to a large and significant reduction in TG (Feng et al., 2024). Recent evidence further supports this interpretation, as a meta-analysis demonstrated that HIIT significantly reduces liver fat content in overweight and obese adults, suggesting its potential to alleviate metabolic complications associated with non-alcoholic fatty liver disease (Khalafi et al., 2022b). Additionally, high baseline TG levels in non-European studies may have contributed to the greater room for improvement.

Regarding BG, HIIT yielded a significant overall reduction, with subgroup analysis again revealing regional variation. The conducted meta-analysis in individuals with metabolic disorders and reported that HIIT significantly reduced BG compared to control groups, with an overall effect size and low heterogeneity, supports overall BG reduction estimate of this research (Serrablo-Torrejon et al., 2020; Cao et al., 2022). European studies showed moderate but consistent reductions with low heterogeneity, while non-European studies showed larger effects accompanied by substantial variability (I^2^ = 83.8%). Although the difference between groups did not reach statistical significance (p = 0.167), the consistency in the European subgroup suggests that baseline glucose regulation, sample size, or intervention monitoring may affect glycemic outcomes (Khalafi et al., 2022a).

Despite these promising results, the analysis revealed several sources of heterogeneity beyond geography. Notably, subgroup analyses based on training frequency and session duration failed to significantly reduce heterogeneity for outcomes such as SBP and HDL-C. This suggests that simple variations in training volume or exposure may not adequately capture the complexity of HIIT response (Luo et al., 2024; Cano-Montoya et al., 2025). Individual variability, such as baseline fitness, medication use, or compliance, likely plays a substantial role, echoing the call from previous work for personalized exercise prescriptions in chronic disease populations (Tjønna et al., 2008; Jelleyman et al., 2015; Mateo-Gallego et al., 2022).

The presence of high risk of bias in nearly one-third of the included studies represents a notable limitation. Trials with inadequate allocation concealment and performance blinding, common in exercise interventions, may have inflated effect estimates due to expectation bias. While our sensitivity analyses confirmed the stability of results, it is plausible that the benefits of HIIT were overestimated in some outcomes. This limitation should temper confidence in the generalizability of the conclusions and highlights the importance of improving methodological rigor in future RCTs.

The quality evaluation of included studies showed generally acceptable methodological rigor. Random sequence generation and attrition bias were well addressed across studies, with 77.3% and 95.5% of studies rated low risk, respectively. However, allocation concealment was poorly reported (only 40.9% low risk), and performance bias was universally high due to it is not feasible to blind participants in exercise trials. This limitation, inherent to behavioral interventions, may inflate effect estimates due to participant expectations or observer effects. Similarly, selective reporting bias was present in a small subset of studies but not widespread, and publication bias was not apparent based on funnel plot analyses.

The results of the sensitivity analyses strengthen the validity of our meta-analytic findings by demonstrating that the observed effects of HIIT on HDL-C, TG, and BG are not unduly influenced by any single study. This robustness is particularly important given the high heterogeneity observed in the initial analyses. Previous meta-analyses have emphasized the variability in HIIT protocols and study populations as key contributors to heterogeneity in cardiometabolic outcomes (Weston et al., 2014; Keating et al., 2017). Despite these variations, the consistent results across the leave-one-out analyses suggest that the beneficial effects of HIIT, particularly improvements in lipid metabolism and glycemic control, are broadly reproducible across different settings and populations.

The relatively stable null finding for SBP further aligns with mixed results reported in earlier systematic reviews, which have shown inconsistent effects of HIIT on blood pressure among clinical populations (Cornelissen and Smart, 2013). This may reflect differences in baseline blood pressure levels, medication use, or the duration and intensity of HIIT interventions. Overall, the robustness demonstrated in our sensitivity analysis provides additional confidence in the reliability of HIIT as a non-pharmacological intervention for improving selected cardiometabolic parameters in MAEP with chronic diseases. Future research should continue to explore how individual characteristics and program variables moderate the response to HIIT, using standardized protocols and long-term follow-up.

### 4.1 Strengths and limitations

This meta-analysis has several notable strengths. First, it presents a comprehensive synthesis of 21 RCTs conducted across four continents, examining the effects of HIIT on five key cardiometabolic outcomes: HDL-C, SBP, DBP, TG, and BG. The inclusion of only RCTs enhances the internal validity of the findings, while the use of a PRISMA-guided search strategy and Cochrane-based risk of bias assessment increases methodological rigor and transparency. Furthermore, subgroup analyses based on geographic region and training duration helped to explain a substantial portion of the initial heterogeneity in outcomes such as SBP, HDL-C, TG, and BG, offering novel insights into potential effect modifiers. In addition, leave-one-out sensitivity analyses confirmed the robustness of the pooled results for HDL-C, TG, and BG, as no single study substantially altered the overall findings. These results enhance confidence in the observed benefits of HIIT for selected cardiometabolic markers in middle-aged and elderly populations with chronic diseases.

However, several limitations should be acknowledged. Despite the use of subgroup and sensitivity analyses, moderate to high residual heterogeneity remained for certain outcomes, particularly TG and HDL-C, which may affect the precision and generalizability of the pooled estimates. Variability in HIIT protocols across studies, including differences in interval duration, frequency, work-to-rest ratios, intensity definitions, and supervision, precludes drawing definitive conclusions regarding optimal training parameters or dose-response relationships. Most included interventions were relatively short-term (≤24 weeks), limiting our understanding of long-term sustainability and clinically meaningful endpoints such as cardiovascular events, hospitalization, or medication reduction. Moreover, although the included studies encompassed geographically diverse populations, most were conducted in European cohorts, and many failed to report critical demographic information such as race or ethnicity, socioeconomic status, and concurrent medication use. Consequently, the generalizability of the findings to broader global populations remains uncertain. The pooled evidence is therefore likely to predominantly reflect European settings, and caution is warranted when extrapolating these results to populations with different cultural, genetic, or environmental backgrounds. Although this research’s primary population of interest was middle-aged and elderly adults, it was unable to conduct age-stratified subgroup analyses due to inconsistent reporting of results by age group in the included studies. This absence represents a limitation, as physiological responses to HIIT may differ between middle-aged and older individuals. Future trials should report age-specific outcomes to enable more nuanced synthesis.

Additionally, this review did not include other relevant physiological markers such as insulin resistance, insulin sensitivity, inflammatory biomarkers (e.g., C-reactive protein), LDL-C, and cardiorespiratory fitness (e.g., VO_2_max), which are important indicators of cardiometabolic health and may mediate or moderate the effects of HIIT. Future studies should aim to incorporate these outcomes to provide a more comprehensive evaluation of HIIT’s mechanisms and clinical implications (HIIT can be integrated as a complementary therapy alongside pharmacological treatment for patients with chronic diseases. In community or rehabilitation settings, structured HIIT may provide an efficient means to improve cardiometabolic health in middle-aged and elderly populations, particularly where resources are constrained). Long-term, large-scale, and demographically inclusive trials are also needed to further elucidate the sustained impact of HIIT on cardiometabolic health in middle-aged and elderly populations with chronic diseases.

## 5 Conclusion

In summary, this meta-analysis provides evidence that HIIT is a generally safe and effective intervention for improving key cardiometabolic outcomes, including HDL-C, TG, BG, and DBP, in MAEP with chronic diseases. However, the effect on SBP was modest and inconsistent. Subgroup analyses revealed that factors such as geographic region and intervention duration significantly influenced the magnitude and consistency of these outcomes, suggesting that contextual variables must be considered when designing and implementing HIIT programs.

Despite the observed benefits, substantial heterogeneity was noted across studies, particularly in intervention protocols. Variations in session frequency (ranging from 2 to 3 times per week), duration (from 3 to 24 weeks), work-to-rest ratios, supervision, and intensity thresholds limit the ability to define a universally optimal HIIT regimen. This underscores the urgent need for more standardized and clearly reported protocols in future trials to facilitate comparability and translation into practice. Additionally, important cardiometabolic markers such as LDL-C, insulin sensitivity, inflammatory biomarkers (e.g., CRP), and cardiorespiratory fitness (e.g., VO_2_max) were not consistently assessed across studies and should be incorporated into future evaluations to provide a more comprehensive understanding of HIIT’s physiological effects.

From a practical perspective, the findings hold actionable implications for clinicians, exercise professionals, and public health policymakers. Specifically, HIIT protocols conducted over 0–8 and 17–24 weeks appear most effective for improving HDL-C, while interventions in European settings demonstrated more consistent benefits for TG and BG. Given its time-efficient nature, HIIT can serve as either a standalone intervention or as a supplement to traditional aerobic or resistance training for patients with chronic diseases. In clinical or community-based settings, short, structured sessions using bodyweight exercises, cycling, or brisk walking, supervised or semi-supervised, may be both feasible and scalable. However, practitioners should tailor HIIT prescriptions to individual fitness levels, comorbidities, and local cultural preferences to enhance adherence and outcomes.

In conclusion, while HIIT shows considerable promise as a non-pharmacological tool in chronic disease management, future research should focus on long-term sustainability, standardized program design, and population-specific implementation strategies to maximize its clinical and public health impact.

## Data Availability

The original contributions presented in the study are included in the article/Supplementary Material, further inquiries can be directed to the corresponding author.

## Supplementary material

SUPPLEMENTARY TABLE 1 PRISMA 2020 Checklist.

SUPPLEMENTARY MATERIAL 1 Exported data from Cochrane Library.

SUPPLEMENTARY MATERIAL 2 Exported data from Embase.

SUPPLEMENTARY MATERIAL 3 Exported data from Web of Science.

SUPPLEMENTARY MATERIAL 4 Exported data from PubMed.

## References

[B1] Álvarez C. Ramírez-Campillo R. Ramírez-Vélez R. Martínez C. Castro-Sepúlveda M. Alonso-Martínez A. (2018). Metabolic effects of resistance or high-intensity interval training among glycemic control-nonresponsive children with insulin resistance. Int. J. Obes. 42 (1), 79–87. 10.1038/ijo.2017.177 28757639

[B48] Aristizabal J. C. Montoya E. Sánchez Y. L. Yepes-Calderón M. Narvaez-Sanchez R. Gallo-Villegas J. A. (2021). Effects of low-volume, high-intensity interval training compared with continuous training on regional and global body composition in adults with metabolic syndrome: a post hoc analysis of a randomized clinical trial. Ann. Nutr. Metabolism 77 (5), 279–288. 10.1159/000518909 34763335

[B2] Batacan R. B. Duncan M. J. Dalbo V. J. Tucker P. S. Fenning A. S. (2017). Effects of high-intensity interval training on cardiometabolic health: a systematic review and meta-analysis of intervention studies. Br. J. sports Med. 51 (6), 494–503. 10.1136/bjsports-2015-095841 27797726

[B3] Bloom D. E. Somnath C. Paul K. Lloyd-Sherlock P. McKee M. Rechel B. (2015). Macroeconomic implications of population ageing and selected policy responses. Lancet 385 (9968), 649–657. 10.1016/s0140-6736(14)61464-1 25468167 PMC4469267

[B4] Borenstein M. Hedges L. V. Higgins J. P. T. Rothstein H. R. (2021). Introduction to meta-analysis. Wiley.

[B5] Cano-Montoya J. Hurtado N. Vergara C. N. Báez Vargas S. Rojas-Vargas M. Martínez-Huenchullán S. (2025). Interindividual variability response to resistance and high-intensity interval training on blood pressure reduction in hypertensive older adults. J. Cardiovasc. Dev. Dis. 12 (1), 30. 10.3390/jcdd12010030 39852308 PMC11765815

[B6] Cao M. Li S. Tang Y. Zou Y (2022). A meta-analysis of high-intensity interval training on glycolipid metabolism in children with metabolic disorders. Front. Pediatr. 10, 887852. 10.3389/fped.2022.887852 35633975 PMC9133662

[B7] Chandler J. Cumpston M. Li T. Page M. J. Welch VJHW (2019). Cochrane handbook for systematic reviews of interventions. Hoboken: Wiley, 4.

[B8] Cohen J. (1960). A coefficient of agreement for nominal scales. Educ. Psychol. Meas. 20 (1), 37–46. 10.1177/001316446002000104

[B9] Collaboration C. (2012). Table 8.5. A: the cochrane collaboration’s tool for assessing risk of bias. Available online at: https://handbook-5-1.cochrane.org/chapter_8/table_8_5_a_the_cochrane_collaborations_tool_for_assessing.htm (Accessed June 4, 2025). 10.1136/bmj.d5928 PMC3196245 22008217

[B10] Collaboration C. (2020). Review Manager (Revman)[Computer Program] the Cochrane Collaboration. London, UK.

[B11] Cornelissen V. A. Smart N. A. (2013). Exercise training for blood pressure: a systematic review and meta‐analysis. J. Am. Heart Assoc. 2 (1), e004473. 10.1161/JAHA.112.004473 23525435 PMC3603230

[B55] Da Silva M. A. R. Baptista L. C. Neves R. S. De França E. Loureiro H. Lira F. S. (2020). The effects of concurrent training combining both resistance exercise and high-intensity interval training or moderate-intensity continuous training on metabolic syndrome. Front. physiology 11, 572. 10.3389/fphys.2020.00572 32595518 PMC7300209

[B49] de Matos D. G. de Almeida-Neto P. F. Moreira O. C. de Souza R. F. Tinoco Cabral B. G. A. Chilibeck P. (2021). Two weekly sessions of high-intensity interval training improve metabolic syndrome and hypertriglyceridemic waist phenotype in older adults: a randomized controlled trial. Metabolic Syndrome Relat. Disord. 19 (6), 332–339. 10.1089/met.2020.0136 33761288

[B12] Edwards J. J. Griffiths M. Deenmamode A. H. P. O’Driscoll J. M. (2023). High-intensity interval training and cardiometabolic health in the general population: a systematic review and meta-analysis of randomised controlled trials. Sports Med. 53 (9), 1753–1763. 10.1007/s40279-023-01863-8 37204620

[B13] Feng J. Zhang Q. Chen B. Chen J. Wang W. Hu Y. (2024). Effects of high-intensity intermittent exercise on glucose and lipid metabolism in type 2 diabetes patients: a systematic review and meta-analysis. Front. Endocrinol. 15, 1360998. 10.3389/fendo.2024.1360998 38978627 PMC11229039

[B14] Ference B. A. Henry N. G. Graham I. Ray K. K. Packard C. J. Bruckert E. (2017). Low-density lipoproteins cause atherosclerotic cardiovascular disease. 1. Evidence from genetic, epidemiologic, and clinical studies. A consensus statement from the european atherosclerosis society consensus panel. Eur. Heart J. 38 (32), 2459–2472. 10.1093/eurheartj/ehx144 28444290 PMC5837225

[B50] Gallo-Villegas J. Castro-Valencia L. A. Pérez L. Restrepo D. Guerrero O. Cardona S. (2022). Efficacy of high-intensity interval-or continuous aerobic-training on insulin resistance and muscle function in adults with metabolic syndrome: a clinical trial. Eur. J. Appl. Physiology 122 (2), 331–344. 10.1007/s00421-021-04835-w 34687360

[B51] Gao K. Su Z. Meng J. Yao Y. Li L. G. Su Y. (2024). Effect of exercise training on some anti-inflammatory adipokines, high sensitivity C-reactive protein, and clinical outcomes in sedentary adults with metabolic syndrome. Biol. Res. Nurs. 26 (1), 125–138. 10.1177/10998004231195541 37579279

[B15] Gibala M. J. Little J. P. MacDonald M. J. Hawley J. A. (2012). Physiological adaptations to low‐volume, high‐intensity interval training in health and disease. J. Physiology 590 (5), 1077–1084. 10.1113/jphysiol.2011.224725 22289907 PMC3381816

[B16] Hannan A. L. Hing W. Simas V. Climstein M. Coombes J. S. Jayasinghe R. (2018). High-intensity interval training Versus moderate-intensity continuous training within cardiac rehabilitation: a systematic review and meta-analysis. Open access J. Sports Med. 9, 1–17. 10.2147/OAJSM.S150596 29416382 PMC5790162

[B52] Heiston E. M. Eichner N. Z. Gilbertson N. M. Malin S. K . (2020). Exercise improves adiposopathy, insulin sensitivity and metabolic syndrome severity independent of intensity. Exp. Physiol. 105 (4), 632–640. 10.1113/EP088158 32020676

[B17] Higgins J. P. T. Green S. (2011). Review: cochrane handbook for systematic reviews for interventions, version 5.1.0, published 3/2011. Julian P.T. higgins and Sally Green, editors. Res. Synthesis Methods 2 (2), 126–130. 10.1002/jrsm.38

[B18] Higgins J. P. T. Thompson S. G. Deeks J. J. Altman D. G. (2003). Measuring inconsistency in meta-analyses. bmj 327 (7414), 557–560. 10.1136/bmj.327.7414.557 12958120 PMC192859

[B19] Higgins J. P. Altman D. G. Sterne J. A. C. (2011). “The cochrane collaboration’s tool for assessing risk of bias,” in Cochrane handbook for systematic reviews of interventions. Editors Higgins J. P. Green S. , 194–202.

[B20] Higgins J. P. T. Thomas J. Chandler J. (2021). Cochrane handbook for systematic reviews of interventions; version 6.2; the cochrane collaboration. London, UK.

[B21] Jelleyman C. Yates T. O'Donovan G. Gray L. J. King J. A. Khunti K. (2015). The effects of high‐intensity interval training on glucose regulation and insulin resistance: a meta‐analysis. Obes. Rev. 16 (11), 942–961. 10.1111/obr.12317 26481101

[B22] Keating S. E. Johnson N. A. Mielke G. I. Coombes J. S. (2017). A systematic review and meta‐analysis of interval training Versus moderate‐intensity continuous training on body adiposity. Obes. Rev. 18 (8), 943–964. 10.1111/obr.12536 28513103

[B23] Khalafi M. Symonds M. E. (2021). The impact of high intensity interval training on liver fat content in overweight or Obese adults: a meta-analysis. Physiology and Behav. 236, 113416. 10.1016/j.physbeh.2021.113416 33823178

[B24] Khalafi M. Ravasi A. A. Malandish A. Rosenkranz S. K. (2022a). The impact of high-intensity interval training on postprandial glucose and insulin: a systematic review and meta-analysis. Diabetes Res. Clin. Pract. 186, 109815. 10.1016/j.diabres.2022.109815 35271876

[B25] Khalafi M. Sakhaei M. H. Kazeminasab F. Symonds M. E. Rosenkranz S. K. (2022b). The impact of high-intensity interval training on vascular function in adults: a systematic review and meta-analysis. Front. Cardiovasc. Med. 9, 1046560. 10.3389/fcvm.2022.1046560 36465439 PMC9713318

[B26] Li X Gao M. Jiao H. (2025). Comparative efficacy of various mind-body exercise types on cardiometabolic health in patients with type 2 diabetes: a network meta-analysis of randomized controlled trials. BMC Cardiovasc. Disord. 25 (1), 291. 10.1186/s12872-025-04745-1 40247204 PMC12004840

[B27] Luo P. Wu R. Gao W. Yan W. Wang R. Ye Y. (2024). Effects of high-intensity interval exercise on arterial stiffness in individuals at risk for cardiovascular disease: a meta-analysis. Front. Cardiovasc. Med. 11, 1376861. 10.3389/fcvm.2024.1376861 38694567 PMC11061535

[B28] Maillard F. Pereira B. Nathalie B. (2018). Effect of high-intensity interval training on total, abdominal and visceral fat mass: a meta-analysis. Sports Med. 48, 269–288. 10.1007/s40279-017-0807-y 29127602

[B29] Mateo-Gallego R. Madinaveitia-Nisarre L. Giné-Gonzalez J. María Bea A. Guerra-Torrecilla L. Baila-Rueda L. (2022). The effects of high-intensity interval training on glucose metabolism, cardiorespiratory fitness and weight control in subjects with diabetes: systematic review a meta-analysis. Diabetes Res. Clin. Pract. 190, 109979. 10.1016/j.diabres.2022.109979 35780905

[B30] Milanović Z. Sporiš G. Weston M. (2015). Effectiveness of high-intensity interval training (Hit) and continuous endurance training for Vo2max improvements: a systematic review and meta-analysis of controlled trials. Sports Med. 45 (10), 1469–1481. 10.1007/s40279-015-0365-0 26243014

[B31] O'Brien MYLES W. Jarrett A. J. Robinson S. A. Bungay A. Mekary SAID Kimmerly D. S. (2020). Impact of high-intensity interval training, moderate-intensity continuous training, and resistance training on endothelial function in older adults. Med. Sci. Sports Exerc. 52 (5), 1057–1067. 10.1249/MSS.0000000000002226 31876667

[B32] Page M. J. McKenzie J. E. Bossuyt P. M. (2021). The prisma 2020 statement: an updated guideline for reporting systematic reviews, 372. 10.1136/bmj.n71 PMC8005924 33782057

[B33] Racil G. Chelly M.-S. Coquart J. Padulo J. Teodor D. F. Russo L. (2023). Long-and short-term high-intensity interval training on lipid profile and cardiovascular disorders in obese Male adolescents. Children 10 (7), 1180. 10.3390/children10071180 37508677 PMC10378083

[B53] Ramirez-Jimenez M. Morales-Palomo F. Ortega J. F. Moreno-Cabañas A. Prada V. G. de Alvarez-Jimenez L. (2020). Effects of exercise training during christmas on body weight and cardiometabolic health in overweight individuals. Int. J. Environ. Res. Public Health 17 (13), 4732. 10.3390/ijerph17134732 32630214 PMC7369896

[B34] Ramos J. S. Dalleck L. C. Erik Tjonna A. Beetham K. S. Coombes J. S. (2015). The impact of high-intensity interval training Versus moderate-intensity continuous training on vascular function: a systematic review and meta-analysis. Sports Med. 45, 679–692. 10.1007/s40279-015-0321-z 25771785

[B54] Ramos J. S. Dalleck L. C. Ramos M. V. Borrani F. Roberts L. Gomersall S. (2016). 12 min/week of high-intensity interval training reduces aortic reservoir pressure in individuals with metabolic syndrome: a randomized trial. J. Hypertens. 34 (10), 1977–1987. 10.1097/HJH.0000000000001034 27467767

[B35] Raudenbush S. W. (1984). Magnitude of teacher expectancy effects on Pupil Iq as a function of the credibility of expectancy induction: a synthesis of findings from 18 experiments. J. Educ. Psychol. 76 (1), 85–97. 10.1037/0022-0663.76.1.85

[B36] Rosenthal R. Jacobson L. (1968). Pygmalion in the classroom. Urban Rev. 3 (1), 16–20. 10.1007/bf02322211

[B37] Seals D. R. Justice J. N. LaRocca T. J. (2016). Physiological geroscience: targeting function to increase healthspan and achieve optimal longevity. J. Physiology 594 (8), 2001–2024. 10.1113/jphysiol.2014.282665 25639909 PMC4933122

[B38] Serrablo-Torrejon I. Lopez-Valenciano A. Ayuso M. Horton E. Mayo X. Medina-Gomez G. (2020). High intensity interval training exercise-induced physiological changes and their potential influence on metabolic syndrome clinical biomarkers: a meta-analysis. BMC Endocr. Disord. 20, 167–12. 10.1186/s12902-020-00640-2 33172413 PMC7653723

[B39] Shuster J. J. (2011). Cochrane handbook for systematic reviews for interventions, version 5.1. 0, published 3/2011. Julian Pt higgins and sally green, editors. Wiley Online Library.

[B40] Solera-Martinez M. Herraiz-Adillo A. Manzanares-Dominguez I. De La Cruz L. L. Martinez-Vizcaino V. Pozuelo-Carrascosa D. P. (2021). High-intensity interval training and cardiometabolic risk factors in children: a meta-analysis. Pediatrics 148 (4), e2021050810. 10.1542/peds.2021-050810 34497117

[B41] Sterne J. A. C. (2009). Meta-analysis in stata: an updated collection from the stata journal. StataCorp LLC.

[B42] Tjønna A. E. Lee S. J. Rognmo Ø. Stølen T. O. Bye A. Haram P. M. (2008). Aerobic interval training *versus* continuous moderate exercise as a treatment for the metabolic syndrome: a pilot study. Circulation 118 (4), 346–354. 10.1161/CIRCULATIONAHA.108.772822 18606913 PMC2777731

[B43] Weston K. S. Wisløff U. Coombes J. S. (2014). High-intensity interval training in patients with lifestyle-induced cardiometabolic disease: a systematic review and meta-analysis. Br. J. Sports Med. 48 (16), 1227–1234. 10.1136/bjsports-2013-092576 24144531

[B44] Wewege M. Van Den Berg R. Ward R. E. Keech A. (2017). The effects of high‐intensity interval training Vs. moderate‐intensity continuous training on body composition in overweight and Obese adults: a systematic review and meta‐analysis. Obes. Rev. 18 (6), 635–646. 10.1111/obr.12532 28401638

[B46] WHO (2025). Ageing: global population. Available online at: https://www.who.int/news-room/questions-and-answers/item/population-ageing (Accessed June 2, 2025).

[B47] Yue T. Wang Y. Liu H. Kong Z. Qi F. (2022). Effects of high-intensity interval Vs. moderate-intensity continuous training on cardiac rehabilitation in patients with cardiovascular disease: a systematic review and meta-analysis. Front. Cardiovasc. Med. 9, 845225. 10.3389/fcvm.2022.845225 35282360 PMC8904881

